# Axial myopia, a protective factor for diabetic retinopathy-role of vascular endothelial growth factor

**DOI:** 10.1038/s41598-022-11220-w

**Published:** 2022-05-05

**Authors:** Ashish Kulshrestha, Nirbhai Singh, Bruttendu Moharana, Parul Chawla Gupta, Jagat Ram, Ramandeep Singh

**Affiliations:** 1 Department of Ophthalmology, Advanced Eye Centre, Post Graduate Institute of Medical Education and Research, Chandigarh, India; 2 Department of Ophthalmology, All India Institute of Medical Sciences, Bhopal, India

**Keywords:** Endocrinology, Risk factors

## Abstract

Long axial length is one of the ocular protective factors in development of diabetic retinopathy (DR). In this study we examined the effect of axial length (AL) on aqueous humor vascular endothelial growth factor (VEGF) levels in patients with diabetes mellitus with or without DR. Forty-eight eyes of 48 participants were divided into three groups of 16 each. Group A consisted of non-diabetic patients, Group B had diabetic patients without DR, and Group C had diabetic patients with treatment-naive non-proliferative DR (NPDR). The groups were further subdivided based on axial lengths i.e., AL ≤ 23.30 mm (A1, B1, C1) and AL > 23.30 mm (A2, B2, C2). Undiluted aqueous humor was obtained during cataract surgery to measure the VEGF levels. We observed significant decrease in VEGF concentration in patients with AL ≥ 23.30 mm as compared with AL ≤ 23.30 mm in non-diabetic as well as diabetic patients. As the eye elongates, there is less secretion of VEGF in non-diabetics as well in diabetics with or without DR. Our findings strengthened the concept that an increase in AL leads to less VEGF in diabetic eyes, thus leading to less severe DR changes.

## Introduction

Diabetic Retinopathy (DR) is considered one of the most important microvascular complications of diabetes mellitus (DM) leading to moderate to severe vision loss^1^. As DR affects mostly the working-class population, it poses an enormous burden to society and the healthcare system. Identification of the factors which might impact the disease pattern is vital for improving DR management. The risk of the onset of retinopathy and its progression is modified by a variety of ocular factors such as optic atrophy, myopia, and glaucoma^2–5^. Previous research has suggested that myopia has a protective effect against DR^5–7^. Vascular endothelial growth factor (VEGF) is an essential pathogenic factor in the development of complications of DR. Several studies have reported a negative correlation between the intraocular concentration of VEGF and the axial length (AL) in subjects without retinal diseases^8–11^. Various other cytokine levels in the aqueous humor of eyes with diabetic retinopathy also show a negative correlation to the AL^12^. The VEGF levels in the anterior chamber reflect the VEGF activity in the retina despite having a lower concentration in aqueous as compared to the vitreous humor^13,14^. In this study, we measured the VEGF concentrations in the aqueous humor of patients with DR and analyzed the relationship between the VEGF concentration and the AL.

## Materials and methods

### Study population

This is a single centre, consecutive, prospective, cross-sectional study of 48 Asian Indian patients above 40 years of age with type II DM. Written informed consents were obtained from the subjects. The study was approved by the Institutional Ethics Committee of the Post Graduate Institute of Medical Education and Research (INT/IEC/2017/361) and adhered to the tenets of the declaration of Helsinki. Patients were excluded, if they had signs of any other retinal or optic nerve pathologies, mature cataract or significant media opacities obstructing retinal evaluation, posterior staphyloma, non-dilating pupil, and untreated or treated proliferative diabetic retinopathy in either eye.

**Figure 1:**
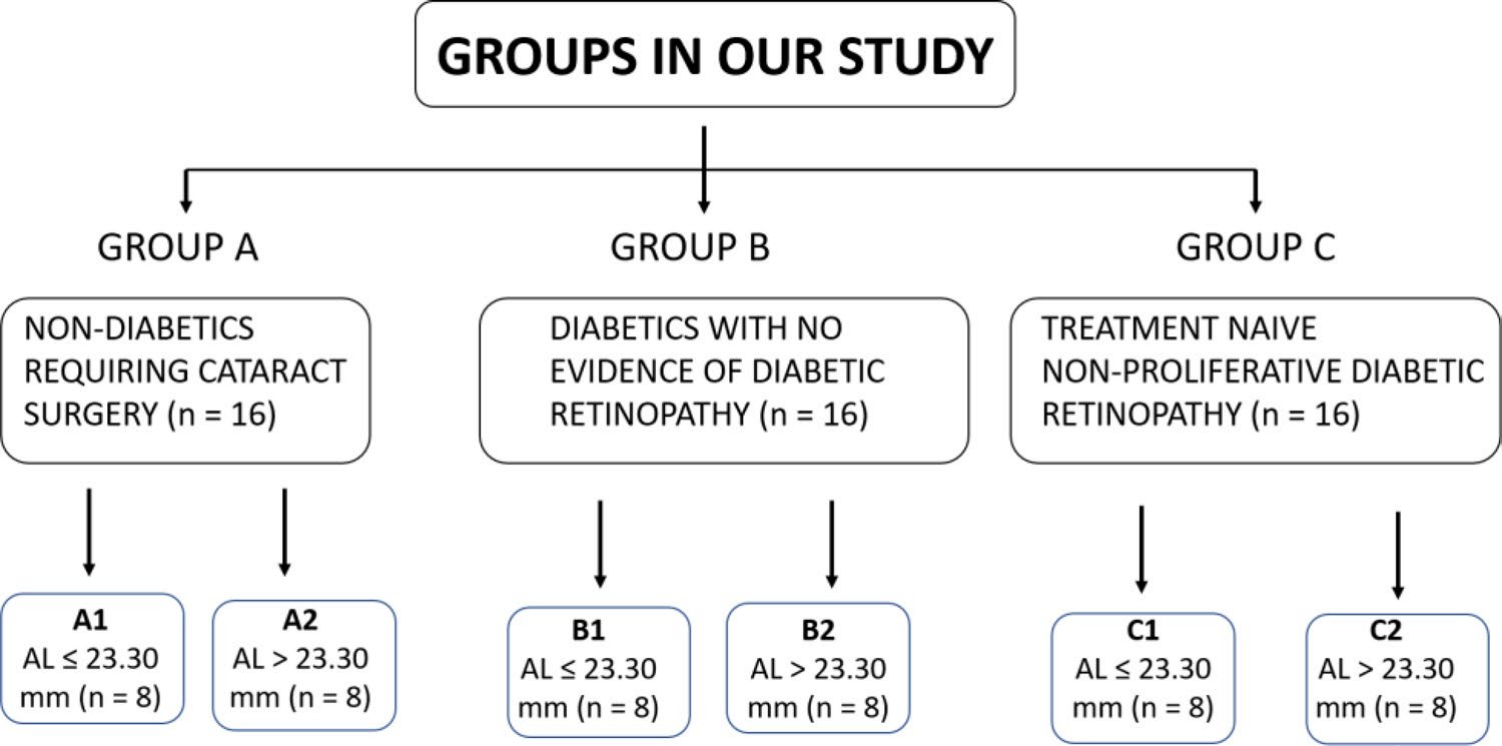
shows allocation of patients into various groups based on axial length and status of diabetic retinopathy.

Patients were divided into three groups, each containing 16 subjects. Group A included patients requiring cataract surgery but without the presence of DM. Group B had patients with DM but without DR who required cataract surgery. Group C had patients with treatment-naive non-proliferative diabetic retinopathy (NPDR) undergoing cataract surgery. Patients with only senile cataract are included in this study. Each group was divided into two subgroups based on their AL. (Fig. 1).

Demographic details, systemic history, and any treatment history were recorded. All patients underwent detailed ocular examination including Snellen’s best-corrected visual acuity, intraocular pressure, slit-lamp biomicroscopy for anterior segment examination, posterior segment examination using slit-lamp biomicroscopy with + 90 D lens, and indirect ophthalmoscopy with + 20D lens.

### Assessment of diabetic retinopathy and axial length

Diabetic retinopathy was classified by the Early Treatment Diabetic Retinopathy Study severity scale^15^. The axial length was measured using the intraocular lens (IOL) master unit (Carl Zeiss, Germany). The mean of the three consecutive readings was taken to ensure consistency and all of them were within 0.02 mm of each other with a sound to noise ratio (SNR) of at least 2.0. The eyes with AL > 23.30 mm were considered as myopic^16^.

### Sample collection and measurement of VEGF levels

Undiluted samples of aqueous humor were obtained at the time of cataract surgery. In six patients of group C, comprising of NPDR with macular edema, the aqueous samples were obtained at the time of anti-VEGF injection. The samples were stored in a freezer at – 800 °C until analysis. Analysis for the VEGF level was done with the commercially available Human growth factor panel (13plex) multi-analyte flow assay kit, LEGENDplex™ panel (VEGF) (Biolegend Inc San Diego, CA, USA). Samples were acquired on BD FACS LSR Fortessa flow cytometer equipped with FACS DIVA 7.0 software (BD Biosciences, CA, USA). FCS files were exported, and data was analyzed using LEGENDplex™ Data analysis software. The sensitivity (Minimum detection concentration) for the detection of Human VEGF was 9.9 pg/ml by using LEGENDplex™ Human Growth Factor Panel (13-plex) Cat. No. 740180. The Flow-based kit LEGENDplex™ Human Growth Factor Panel as per the kit literature provided is highly specific and does not show any cross-reactivity with other recombinant proteins. VEGF pair can detect both VEGF165 and VEGF121.

The primary outcome of the study was to evaluate the relationship between VEGF concentration in aqueous humor and AL of the globe in diabetic and non-diabetic patients. As the secondary outcome, we also compared the VEGF concentration between diabetics, with or without DR, and non-diabetics.

### Statistical analysis

Statistical analysis was performed with SPSS (Statistical Package for Social Sciences) version 20.0 for Windows (Microsoft, Armonk, NY, USA). The obtained data were tested for normal distribution using the Shapiro–Wilk test. Data distribution was described in the form of mean and standard deviation. Unpaired Student's t-test was applied to compare the two group means. Both inter and intragroup comparisons were made. For multiple group comparisons, the ANOVA test was used. *P* < 0.05 was considered statistically significant.

### Conference presentation

Presented as poster in Annual ARVO meeting at Vancouver, 2019.

## Results

A total of 48 subjects (23 males and 25 females) were recruited. The mean age was 56.87 ± 8.36 years in group A, 60.63 ± 6.81 years in group B and 58.25 ± 11.47 years in group C. The mean VEGF concentration of the three sub-groups in patients with AL ≤ 23.30 mm (A1, B1, C1) were 112.59 ± 30.43 pg/ml, 89.39 ± 16.36 pg/ml, 125.25 ± 44.75 pg/ml, respectively. Similarly, the mean VEGF values of the three sub-groups in patients with AL > 23.30 mm (A2, B2, C2) were 53.92 ± 17.3 pg/ml, 58.50 ± 17.16 pg/ml, 75.95 ± 32.6 pg/ml respectively. (Table 1).

**Table 1:** Axial lengths and corresponding aqueous VEGF levels of different groups and sub-groups.

Groups	Axial lenghth (mm)		VEGF level (pg/ml)	*P* value
	Mean ± SD	Range		
Group A	23.64 ± 2.51	19.55–29.77	83.26 ± 38.60	
A1	21.76 ± 1.23	19.55–22.95	112.59 ± 30.43	0.001
A2	25.52 ± 1.96	24.10–29.77	53.92 ± 17.3	
Group B	23.51 ± 1.45	21.68–27.18	73.95 ± 22.74	
B1	22.54 ± 0.64	21.68–23.14	89.39 ± 16.36	0.002
B2	24.83 ± 1.39	23.33–27.18	58.50 ± 17.16	
Group C	23.46 ± 1.15	22.01–26.51	100.61 ± 45.60	
C1	22.65 ± 0.53	22.01–23.30	125.25 ± 44.75	0.025
C2	24.27 ± 1.02	23.47–26.51	75.95 ± 32.6	

*Group A:* non-diabetic patients (A1 AL ≤ 23.30 mm, A2 AL > 23.30 mm), *Group B*: diabetic patients without diabetic retinopathy (B1 AL ≤ 23.30 mm, B2 AL > 23.30 mm), *Group C*: diabetic patients with treatment naive non-proliferative diabetic retinopathy (C1 AL ≤ 23.30 mm, C2 AL > 23.30 mm).

AL- axial length, mm- millimeters, ml- milliliter, pg- picogram, SD- standard deviation, VEGF- vascular endothelial growth factor.

Unpaired *t* test is used for comparison of intra-group VEGF levels. *P* < 0.05—statistically significant.

In group A (non-diabetics), the mean AL was 23.64 ± 2.51 mm. The mean VEGF in this group was 83.26 ± 38.60 pg/ml. For every 1 mm increase in axial length, the VEGF decreased by 12.04 pg/ml in this group. In group B (Diabetics without NPDR), the mean AL was 23.51 ± 1.45 mm. The mean VEGF in this group was 73.95 ± 22.74 pg/ml. For every 1 mm increase in axial length in this group, there was a decrease in VEGF by 12.04 pg/ml. In group C (Diabetics with NPDR), the mean AL was 23.46 ± 1.15 mm. The mean VEGF in this group was 100.61 ± 45.60 pg/ml. There was a 13.45 pg/ml decrease in VEGF values for each mm increase in axial length in group C.

**Figure 2:**
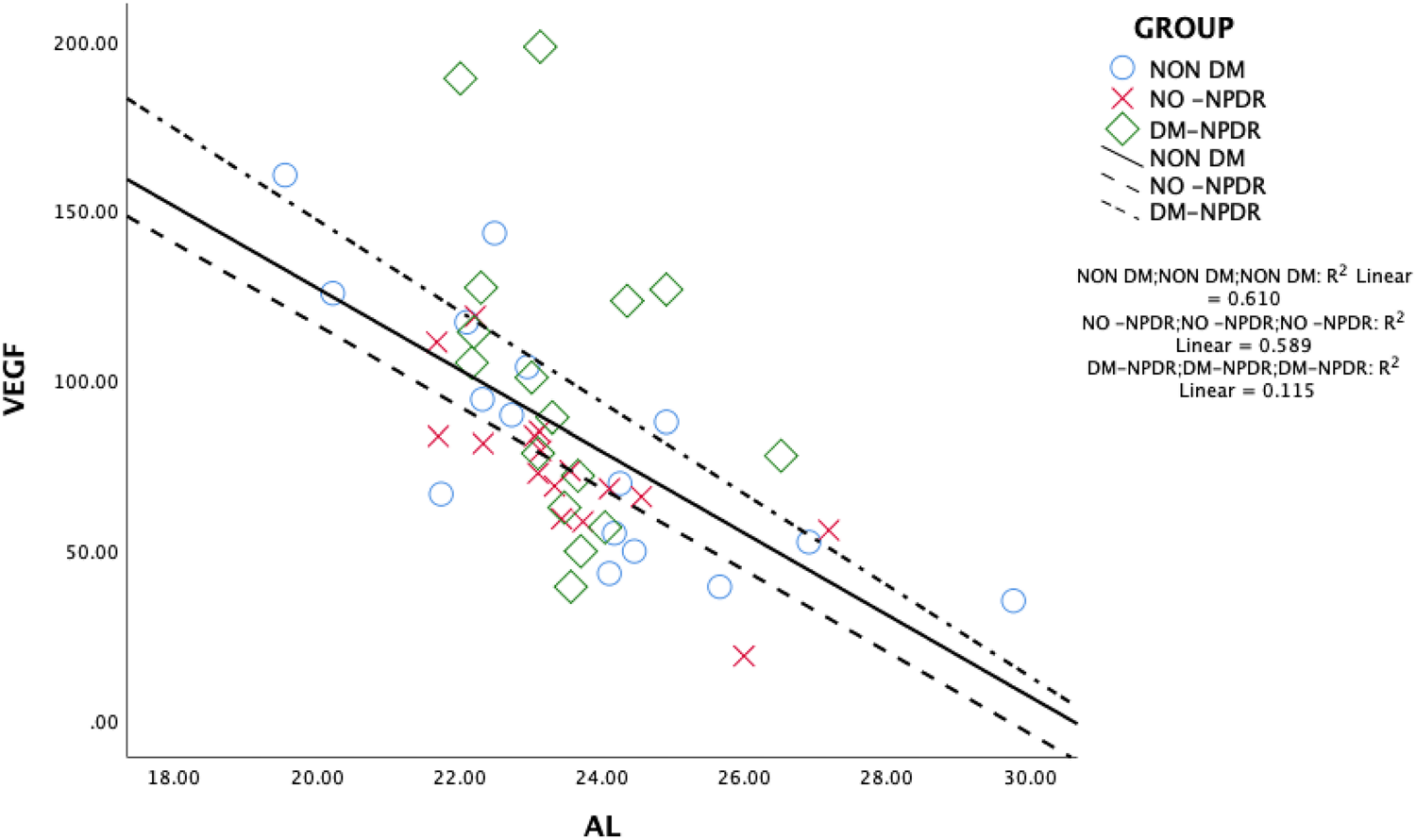
Scatter diagram showing correlation between concentration of vascular endothelial growth factor (VEGF) and axial length.

Figure 2 depicts the scatter diagram correlating the aqueous humor VEGF concentration with the AL across various groups and subgroups. Pearson product moment correlation test was used to evaluate the correlation between the concentration of cytokines in the aqueous humor and AL. In non-diabetics the concentration of VEGF in the aqueous humor correlated negatively with AL (Pearson product moment correlation ρ = − 0.781) and the values were statistically significant (*P* < 0.001). Also in diabetics without NPDR, the aqueous humor VEGF concentration had a negatively correlation with AL (Pearson product moment correlation ρ = − 0.767) and it was statistically significant (*P* = 0.001). In patients with NPDR, although the VEGF concentration in the aqueous humor samples negatively correlated with AL but not as strong as in the previous two groups (Pearson product moment correlation ρ = − 0.339, *P* = 0.199). On applying ANCOVA, the slopes of each linear regression differ from each other significantly (*P* < 0.001) with mean VEGF value estimated to be 85.939 pg/ml. The power of the study was estimated to be 90%.

In sub-group analysis, the mean AL in sub-groups A1 (AL ≤ 23.30 mm) and A2 (AL > 23.30 mm) were 21.76 ± 1.23 mm and 25.52 ± 1.96 mm respectively. The mean VEGF levels were 112.59 ± 30.43 pg/ml and 53.92 ± 17.3 pg/ml in sub-groups A1 and A2 respectively. The difference in VEGF levels was statistically significant (*p* = 0.001). Similarly, the mean AL in sub-groups B1 (AL ≤ 23.30 mm) and B2 (AL > 23.30 mm) were 22.54 ± 0.64 mm and 24.83 ± 1.39 mm respectively. The mean VEGF levels were 89.39 ± 16.36 pg/ml and 58.50 ± 17.16 pg/ml in sub-groups B1 and B2 respectively (p = 0.002). In group C, the mean AL in sub-groups C1 (AL ≤ 23.30 mm) and C2 (AL > 23.30 mm) were 22.65 ± 0.53 mm and 24.27 ± 1.02 mm respectively. The mean VEGF levels were 125.25 ± 44.75 pg/ml and 75.95 ± 32.6 pg/ml in sub-groups C1 and C2 respectively (*p* = 0.025) (Table 1).

In group C, out of 16 patients, 4 had mild NPDR, 7 had moderate NPDR and 5 had severe NPDR. The mean AL (mm) in patients with severe, moderate, and mild NPDR were 23.05 ± 0.84, 23.94 ± 1.30, and 23.14 ± 0.69 respectively. The mean VEGF levels (pg/ml) in eyes with severe, moderate, and mild NPDR were 140.06 ± 49.49, 81.85 ± 26.96, and 84.1 ± 25.08 respectively. Thus, patients with longer axial length had low VEGF levels and less severe form of DR. However, this observation was not statistically significant (Table 2).

**Table 2:** Mean axial length and VEGF values in Group C (diabetics with diabetic retinopathy).

Group C	Number of patients (N = 16)	Axial length (mm)		Mean VEGF levels (pg/ml)
		Mean ± SD	Range	
Mild NPDR	4	23.14 ± 0.69	22.2–23.7	84.1 ± 25.08
Moderate NPDR	7	23.94 ± 1.30	22.17–26.51	81.85 ± 26.96
Severe NPDR	5	23.05 ± 0.84	22.01–24.35	140.07 ± 49.49

NPDR-Non proliferative diabetic retinopathy, VEGF- vascular endothelial growth factor.

## Discussion

The vascular endothelial growth factor is the major cytokine in the pathogenesis of DR showing a strong relationship between the increase in intraocular VEGF levels and the severity of DR^17^. There are various studies, showing the association between long AL and its protective role against DR^5–7^. Wang et al., in their meta-analysis, suspected that longer eyes might have lower VEGF levels, which may prevent the occurrence of DR. They showed that when each millimeter increase in AL was analyzed as a continuous variable for myopia, it was significantly associated with a decreased risk of DR^6^. Pierro et al. showed that considering all the confounding factors, the presence of retinopathy was associated with shorter axial length^18^. Jain et al. in their study observed that myopic refractive error in cases with diabetic retinopathy was always less than 5 diopters, thus concluding that myopia was protective against diabetic retinopathy^3^. Lim et al. observed that long axial length (AL), and deeper anterior chamber depth (ACD) were associated with less likelihood of developing any DR^4^. Our study also demonstrated that longer AL was associated with lesser VEGF in the eye.

Several studies have reported a negative correlation between the intraocular concentration of VEGF and AL in subjects without retinal diseases^8–11^. In this study, we have demonstrated that the VEGF levels in eyes with DR also show a negative trend with axial elongation which may explain the fact that longer eyes are protected against DR due to relatively lower secretion of the VEGF.

We observed a statistically significant decrease (*P* < 0.05) in the levels of VEGF concentration in aqueous humor of patients with AL ≥ 23.30 mm as compared with AL ≤ 23.30 mm in non-diabetic as well as diabetic patients. On further comparing and analysing VEGF concentrations within each subgroups, we also found a statistically significant trend in the decrement of VEGF concentrations as the eye elongates. In non-diabetic subjects, the mean VEGF was 112.59 pg/ml in shorter eyes and 53.93 pg/ml in longer eyes (*P* = 0.001). Similarly, in diabetic patients but without presence of DR, the mean VEGF was 89.398 pg/ml in shorter eyes whereas it was 58.503 pg/ml in longer eyes (*P* = 0.002). In eyes with presence of DR, the mean VEGF levels were 125.256 pg/ml in shorter eyes and 75.959 pg/ml in longer eyes (*P* = 0.025). Above implies that as the eye elongates, there is less secretion of VEGF in non-diabetics as well in diabetics with or without DR.

We also compared the VEGF levels and the AL among the eyes with different severity level of NPDR. The mean AL in patients with moderate and mild NPDR was higher as compared to those with severe NPDR (moderate > mild > severe). The corresponding VEGF level was maximum in severe NPDR group as expected. However, the VEGF level was lower in the moderate NPDR group than the mild NPDR group which may be associated with longer AL in the moderate NPDR group. The intraocular VEGF level increases with increase in severity of DR. However, in this study, the VEGF level in patients with mild and moderate NPDR corresponded to the AL rather than the severity of DR. This highlights the importance of AL in intraocular VEGF level. However, this difference was not statistically significant. There have been no study yet which reports similar findings. This will need further analysis in a larger cohort to establish if such findings have strong statistical support.

The exact mechanisms underlying the protective effect of myopia on DR are currently unclear. Most have centered around the pathologic changes associated with axial globe elongation^19–25^. One possible explanation is that the VEGF in the anterior chamber and the vitreous cavity might be diluted because of longer axial length and therefore, increased intraocular volume^11^. Complete posterior vitreous detachment (PVD) and vitreous syneresis are also more common in myopes. It has been reported that the clearance of growth and permeability factors from the retina is enhanced following vitrectomy due to decreases viscosity after removal of vitreous gel^19^. As PVD increases the fluidity and thereby decreasing the viscosity of the vitreous, it might be associated with a faster turnover of VEGF out of the eye. Chorioretinal thinning, another frequent finding in myopes, also might have a protective effect by reducing the metabolic demand and facilitating the diffusion of oxygen through the retina^19^. The thinning might cause relatively increased choroidal perfusion and decreased retinal hypoxia resulting in decreased VEGF production. Studies have also demonstrated the correlation between neurodegeneration and retinal neuron dysfunction with axial elongation^22,23^. These changes decrease the metabolic demand in the outer retina, which helps to attenuate the effects of hypoxia in diabetics, which might further decrease the VEGF levels in these eyes.

The general limitations of this study include a lower number of participants, which may have led to an increased variance in our results. Also, we assessed the levels of VEGF in aqueous humor and not vitreous humor, which might have affected our cytokine levels although studies have shown that aqueous VEGF levels correlate with the VEGF activity in the retina^13,14^. We also did not have subjects with high axial length and staphyloma. Further study in a larger cohort will help in gaining more insight.

In conclusion, this study provides evidence to support the well-described clinical impression that myopic eyes are less likely to develop DR, particularly more severe stages of DR. This study showed that as the axial length increases, the VEGF levels decrease in both diabetics and non-diabetics. This study contributes further insights into the pathogenetic pathways for DR and may help clinicians in their assessment of the risk of DR in patients with myopia.

## Data Availability

The datasets generated during and/or analysed during the current study are available from the corresponding author on reasonable request.

## References

[1] Klein R, Klein BE, Moss SE (1989). The Wisconsin epidemiological study of diabetic retinopathy: A review. Diabetes Metab. Rev..

[2] Dogru M, Inoue M, Nakamura M, Yamamoto M (1998). Modifying factors related to asymmetric diabetic retinopathy. Eye.

[3] Jain IS, Luthra CL, Das T (1967). Diabetic retinopathy and its relation to errors of refraction. Arch. Ophthalmol..

[4] Lim LS (2010). Are myopic eyes less likely to have diabetic retinopathy?. Ophthalmology.

[5] Man RE (2012). Longer axial length is protective of diabetic retinopathy and macular edema. Ophthalmology.

[6] Wang X (2016). Myopia and diabetic retinopathy: A systematic review and meta-analysis. Diabetes Res. Clin. Pract..

[7] He J (2017). Lens power, axial length-to-corneal radius ratio, and association with diabetic retinopathy in the adult population with type 2 diabetes. Ophthalmology.

[8] Zhu D (2015). Intracameral interleukin 1β, 6, 8, 10, 12p, tumor necrosis factor α and vascular endothelial growth factor and axial length in patients with cataract. PLoS One.

[9] Jonas JB, Tao Y, Neumaier M, Findeisen P (2010). VEGF and refractive error. Ophthalmology.

[10] Hu Q (2015). Intravitreal vascular endothelial growth factor concentration and axial length. Retina.

[11] Sawada O (2011). Negative correlation between aqueous vascular endothelial growth factor levels and axial length. Jpn. J. Ophthalmol..

[12] Hong F (2020). Relationship between aqueous humor levels of cytokines and axial length in patients with diabetic retinopathy. Asia Pac. J. Ophthalmol..

[13] Funatsu H (2005). Aqueous humor levels of cytokines are related to vitreous levels and progression of diabetic retinopathy in diabetic patients. Graefes. Arch. Clin. Exp. Ophthalmol..

[14] Endo M (2001). Increased levels of vascular endothelial growth factor and advanced glycation end products in aqueous humor of patients with diabetic retinopathy. Horm. Metab. Res..

[15] Early Treatment Diabetic Retinopathy Study group (1991). Early photocoagulation for diabetic retinopathy. ETDRS report no.9. Ophthalmology.

[16] Roy, A., Kar, M., Mandal, D., Ray, R. S., Kar, C. Variation of axial ocular dimensions with age, sex, height, BMI-and their relation to refractive status. *J. Clin. Diagn. Res.* **9**, AC01-AC4 (2015). 10.7860/JCDR/2015/10555.5445 PMC4347057 25737966

[17] Selim KM, Sahan D, Muhittin T, Osman C, Mustafa O (2010). Increased levels of vascular endothelial growth factor in the aqueous humor of patients with diabetic retinopathy. Indian J. Ophthalmol..

[18] Pierro L (1999). Axial length in patients with diabetes. Retina.

[19] Stefansson E (2006). Ocular oxygenation and treatment of diabetic retinopathy. Surv. Ophthalmol..

[20] Quigley M, Cohen S (1999). A new pressure attenuation index to evaluate retinal circulation: A link to protective factors in diabetic retinopathy. Arch. Ophthalmol..

[21] Man RE (2014). Decreased retinal capillary flow is not a mediator of the protective myopia-diabetic retinopathy relationship. Invest. Ophthalmol. Vis. Sci..

[22] Luu CD, Lau AM, Lee SY (2006). Multifocal electroretinogram in adults and children with myopia. Arch. Ophthalmol..

[23] Wolsley CJ, Saunders KJ, Silvestri G, Anderson RS (2008). Investigation of changes in the myopic retina using multifocal electroretinograms, optical coherence tomography and peripheral resolution acuity. Vis. Res..

[24] Tagawa H, McMeel JW, Trempe CL (1986). Role of vitreous in diabetic retinopathy II. Active and inactive vitreous changes. Ophthalmology.

[25] Akiba J, Arzabe CW, Trempe CL (1990). Posterior vitreous detachment and neovascularization in diabetic retinopathy. Ophthalmology.

